# Effects of Electrical Stimulation Therapy for Residual Foot Drop Following Lumbar Spinal Stenosis Surgery: A Case Report

**DOI:** 10.7759/cureus.85676

**Published:** 2025-06-10

**Authors:** Hikaru Yanagida, Yuuki Uetani, Tomomi Takimoto

**Affiliations:** 1 Department of Rehabilitation, Jiseikai-Nerimatakanodai Hospital, Tokyo, JPN

**Keywords:** foot drop, functional electrical stimulation (fes), gait, lumbar spinal stenosis (lss), neuromuscular electrical stimulation (nmes)

## Abstract

This case report describes a patient with residual foot drop following lumbar spinal stenosis (LSS) surgery, who demonstrated improvement after undergoing neuromuscular electrical stimulation (NMES) and functional electrical stimulation (FES). The patient, a 70-year-old man, had foot drop attributed to LSS. During the initial phase of treatment, NMES using high-voltage pulsed current was applied to facilitate contraction of the ankle dorsiflexor muscles. As his walking ability stabilized, FES was introduced during ambulation to support functional muscle activation. As a result, the patient experienced improvements in gait function, ankle dorsiflexion function, and fear of walking. This case suggests that in patients with residual foot drop after LSS surgery, a combination of NMES to enhance muscle contraction and FES to assist walking may contribute to functional recovery.

## Introduction

Lumbar spinal stenosis (LSS) is a condition commonly observed in older adults [1]. It is characterized by the narrowing of the spinal canal and neural foramina, which leads to compression of the nerves and blood vessels. As a result, patients often experience symptoms such as lower extremity pain, numbness, muscle weakness, and reduced walking ability, typically accompanied by intermittent claudication. The incidence of foot drop among patients with LSS is relatively low, estimated to be 5% to 12% [2]. However, when foot drop is present, walking ability is further compromised. One report noted that 55.6% of patients with preoperative foot drop continued to experience walking difficulties after surgery, suggesting that foot drop may be a poor prognostic factor in LSS [3]. In cases where walking ability is significantly impaired, LSS can considerably affect patients’ activities of daily living (ADL) and quality of life (QOL) [4].

Electrical stimulation therapy is one treatment option for foot drop. Neuromuscular electrical stimulation (NMES) and functional electrical stimulation (FES) are the primary modalities used. NMES involves applying electrical stimulation to peripheral nerves or motor points of targeted muscles to elicit limb movement [5]. It has been reported to enhance muscle mass and increase maximal muscle strength [6]. In contrast, FES facilitates purposeful and functional movements, such as grasping objects or walking, by inducing muscle contractions through electrical stimulation. FES is expected to contribute to the recovery of voluntary motor function [7]. Several studies have demonstrated the effectiveness of FES in managing foot drop, particularly by improving walking speed and ankle dorsiflexion function [8,9]. However, there are few reports specifically addressing the efficacy of electrical stimulation therapy for foot drop resulting from lumbar spine disorders, with only sporadic case reports currently available [10]. Thus, the effectiveness of electrical stimulation therapy for foot drop caused by LSS remains uncertain. This case report aims to present the therapeutic outcomes of NMES and FES in a patient with residual foot drop following LSS surgery, who exhibited improvements in physical function and walking ability.

## Case presentation

**Figure 1:**
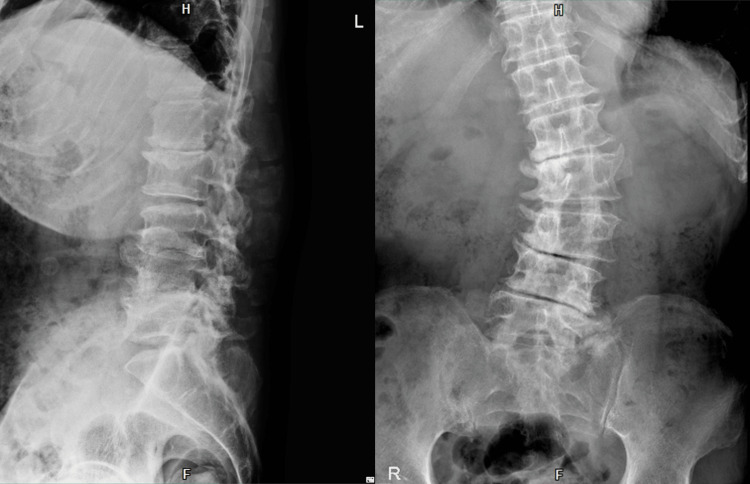
Lumbar spine radiograph at the admission

A 70-year-old man presented with left-sided foot drop attributed to LSS. He first noticed the foot drop two years before surgery; however, since he was still able to walk, surgery was not performed at that time in accordance with his wishes. At that time, the Manual Muscle Testing (MMT) score of the tibialis anterior muscle was 1. Two weeks later, due to worsening walking ability, he returned to the hospital and underwent L2-4 spinous process splitting and L2-5 laminectomy on Day X (the day of surgery). Postoperatively, the foot drop persisted. Although orthotic therapy using an ankle-foot orthosis (AFO) was attempted, he did not use it regularly, indicating poor adherence. He was subsequently admitted to the rehabilitation ward of our hospital on Day X+18. At admission, typical LSS symptoms such as intermittent claudication, lower extremity pain, and sensory deficits (e.g., numbness) were not observed. An X-ray image taken at admission is shown in Figure 1.

The subject of this case report was given a thorough explanation, both verbally and in writing, regarding the purpose of the report and the handling of personal information. Written informed consent was obtained prior to publication. The assessment items were as follows: Gait function was assessed using the 10-Meter Walk Test (time and number of steps) and the Timed Up and Go (TUG) test. Balance was assessed with the Berg Balance Scale (BBS). Muscle strength was assessed using MMT for the left tibialis anterior, extensor hallucis longus, extensor digitorum longus, triceps surae, gluteus maximus, and gluteus medius. Ankle dorsiflexion range of motion was measured for both active (AROM) and passive (PROM) movements. Additionally, fear of walking was assessed using a Visual Analogue Scale (VAS). Calf circumference was measured bilaterally to assess potential muscle atrophy.

**Figure 2:**
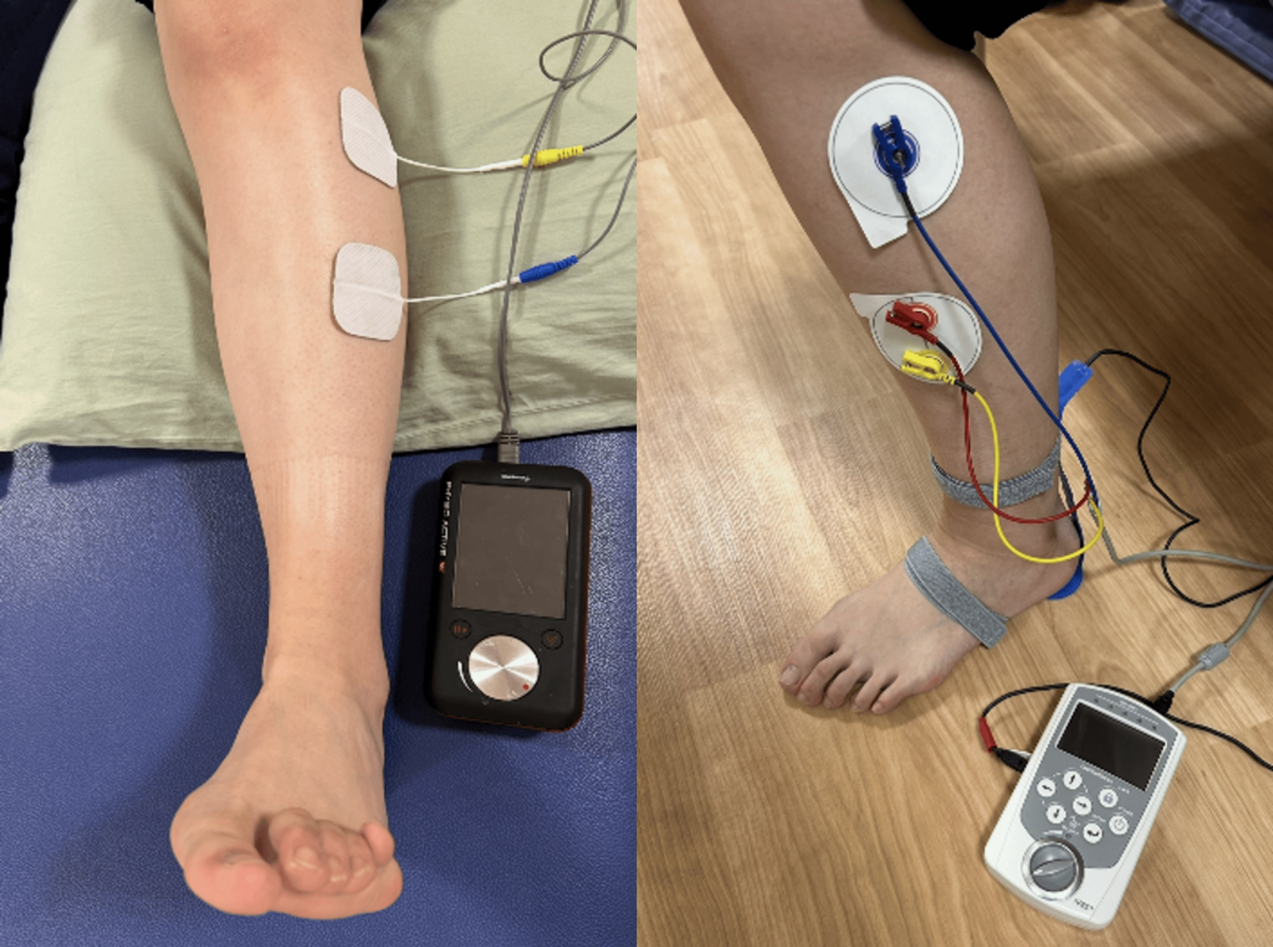
Setups for electrical stimulation therapies The figure shows the electrode placement and equipment setup for NMES (left panel) and FES (right panel)

The treatment methods were as follows. In addition to general rehabilitation, comprising range of motion exercises, muscle strengthening exercises for muscles other than those around the ankle, balance training, gait training, and ADL training, electrical stimulation therapy using NMES and FES was implemented (Figure 2).

For NMES, the PHYSIO ACTIVE HV stimulator (Sakai Medical, Tokyo, Japan), which delivers high-voltage pulsed current (HVPC), was used. Stimulation was applied to the left tibialis anterior and extensor digitorum longus muscles. Parameters were set to a frequency of 30 Hz, a pulse width of 50 μs, and a duty cycle of 1:2, with the off-time longer than the on-time. Stimulation intensity was set to the maximum level tolerable without pain (60-70 V). The patient was positioned supine with the left knee slightly flexed and performed active ankle dorsiflexion in coordination with the stimulation. Each NMES session lasted up to 20 minutes and was performed daily, involving a total of 100 stimulated contractions (repetitions) per session, starting from day X+22. For FES, the sensor-trigger mode of the IVES stimulator (OG Giken, Okayama, Japan) was used. Based on input from a sensor placed on the heel, stimulation was delivered to the peroneal nerve during the swing phase of the left leg to assist ankle dorsiflexion. The stimulation frequency was set to 30 Hz, and intensity was adjusted to the maximum level without discomfort (50-70%). Walking training with FES was performed for approximately 200-400 meters per day, starting from day X+44. No adverse events or complications were observed throughout the course of electrical stimulation therapy, and the treatment was well tolerated by the patient.

Clinical course

Assessment on Admission Day

The 10-Meter Walk Test took 27.1 seconds and 55 steps. The TUG required 28.6 seconds for the right turn and 29.2 seconds for the left turn. The BBS score was 44 points. MMT scores on the left side were as follows: tibialis anterior, 1; extensor hallucis longus, 1; extensor digitorum longus, 3; triceps surae, 2; gluteus maximus, 3; and gluteus medius, 2. Ankle dorsiflexion AROM was -15°, and PROM was 0°. The VAS score for fear of walking was 6.7 cm. Calf circumference was 35 cm on the right and 31 cm on the left, suggesting muscle atrophy on the affected side (Table 1). Regarding gait pattern, left foot drop was observed during the swing phase of the left lower limb, along with reduced foot clearance and marked compensatory movement involving left hip flexion. In addition, stride length was shortened, resulting in a step-to gait pattern (Video 1).

**Table 1:** Changes in assessment outcomes over the rehabilitation period 10mWT: 10-Meter Walk Test, MMT: Manual Muscle Testing, TUG: Timed Up and Go Test, BBS: Berg Balance Scale, ROM: Range of Motion, AROM: Active Range of Motion, PROM: Passive Range of Motion, VAS: Visual Analogue Scale, s: seconds, °: degrees, cm: centimeters.

Assessment Item	Admission Day (Day X+18–21)	Day X+32 (Immediate Effect)	Intermediate (Day X+44)	Discharge (Day X+54)
10mWT (s)	27.1	19.5 → 16.4	17.9	11.2
10mWT (steps)	55	38 → 31	30	22
TUG - Right turn (s)	28.6	-	23.4	18.7
TUG - Left turn (s)	29.2	-	26.5	19.1
BBS (points)	44	-	48	49
MMT (Left side)				
Tibialis anterior	1	1 → 1	1	2
Extensor hallucis longus	1	1 → 1	1	1
Extensor digitorum longus	3	3 → 3	4	4
Triceps surae	2		2	2
Gluteus maximus	3		3	3
Gluteus medius	2		2	2
Ankle Dorsiflexion ROM				
AROM (°)	-15	-15 → -15	-15	-10
PROM (°)	0	0 → 0	0	0
VAS for Fear of Walking (cm)	6.7	5.0 → 4.2	2.1	0.4
Calf Circumference (cm) (R/L)	35 / 31		35 / 31.5	35 / 31.5

**Video 1:** Changes in patient gait during rehabilitation

Initiation of NMES (Day X+22), Assessment of Immediate Effect (Day X+32)

**Figure 3:**
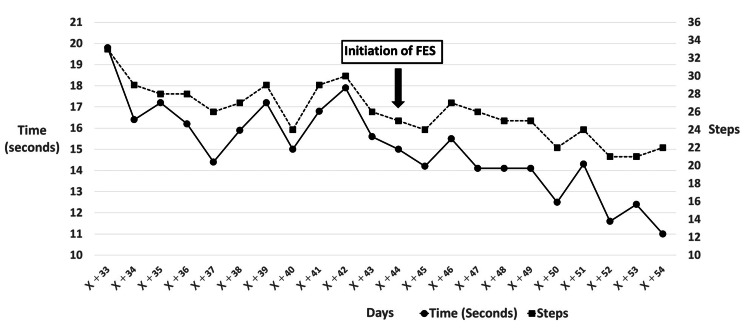
Daily 10-Meter Walk Test performance The figure shows the daily trend of the 10-Meter Walk Test time (left y-axis) and the number of steps (right y-axis) from Day X+33 to Day X+54. The point of FES initiation (Day X+44) is indicated.

On Day X+22, NMES was initiated with the goal of strengthening the tibialis anterior muscle and improving ankle dorsiflexion function. During NMES application, contraction of the tibialis anterior and visible dorsiflexion of the ankle joint were observed. On Day X+32, to assess the immediate effect of NMES, the 10-Meter Walk Test, MMT, and ankle dorsiflexion AROM and PROM were measured before and after NMES application. Immediate improvements were observed in the 10-Meter Walk Test (19.5 s/38 steps → 16.4 s /31 steps) and in the VAS score (5.0 cm → 4.2 cm). However, no changes were noted in MMT or ROM. From Day X+33 onward, to monitor the time-course effect of NMES, it was decided to measure the 10-Meter Walk Test daily before the start of rehabilitation (Figure 3).

Intermediate Assessment and Initiation of FES (Day X+44)

An intermediate assessment was conducted on Day X+44, with the following results. The 10-Meter Walk Test took 17.9 seconds with 30 steps. The TUG required 23.4 seconds for the right turn and 26.5 seconds for the left turn. The BBS score was 48 points. MMT scores on the left side were as follows: tibialis anterior, 1; extensor hallucis longus, 1; extensor digitorum longus, 4; triceps surae, 2; gluteus maximus, 3; and gluteus medius, 2. Ankle dorsiflexion AROM was -15°, and PROM was 0°. The VAS score for fear of walking was 2.1 cm. Calf circumference was 35 cm on the right and 31.5 cm on the left. The 10-Meter Walk Test, measured daily since Day X+32, showed improvement compared to both admission and the start of daily measurements. However, the rate of improvement had gradually declined. In contrast, a marked reduction in fear of walking was observed, and gait stability had improved. The gait pattern also showed a closer approximation to normal gait than at admission. Based on these findings, FES was initiated in addition to ongoing NMES, with the aim of further enhancing walking function.

Assessment at Discharge (Day X+54)

An assessment at discharge was conducted on Day X+54, with the following results. The 10-Meter Walk Test took 11.2 seconds with 22 steps. The TUG required 18.7 seconds for the right turn and 19.1 seconds for the left turn. The BBS score was 49 points. MMT scores on the left side were as follows: tibialis anterior, 2; extensor hallucis longus, 1; extensor digitorum longus, 4; triceps surae, 2; gluteus maximus, 3; and gluteus medius, 2. Ankle dorsiflexion AROM was -10°, and PROM remained at 0°. The VAS score for fear of walking was 0.4 cm. Calf circumference was 35 cm on the right and 31.5 cm on the left. Regarding the gait pattern, although compensatory left hip flexion during the swing phase persisted, improvements in foot clearance and increased stride length were observed. As a result, a reciprocal gait pattern was achieved.

## Discussion

This case report describes a patient with residual foot drop following LSS surgery who was treated with electrical stimulation using NMES and FES. These interventions led to improvement in ankle function, enhanced walking ability, and a reduction in fear of walking. Previous studies have reported the potential for improvement in foot drop caused by lumbar degenerative disease following surgical intervention, highlighting prognostic factors such as the duration of paralysis and preoperative muscle strength as significant influences on recovery outcomes [11]. In the present case, the patient had a preoperative ankle dorsiflexion strength of MMT 1, and two years had passed since the onset of foot drop. Based on these factors, sufficient recovery through surgery alone was considered unlikely. Electrical stimulation therapy is indicated when the peripheral nerve remains intact, in other words, when the lower motor neuron pathway to the target muscle is preserved and effective muscle contraction can be induced [5]. In this case, the presence of visible muscle contraction and joint movement in response to electrical stimulation suggests that surgical decompression may have relieved nerve compression, thereby making electrical stimulation therapy applicable. As discussed above, even in situations where spontaneous recovery is unlikely, this case suggests that electrical stimulation therapy can be a viable treatment option for patients with foot drop.

This section discusses the effects of NMES on ankle function recovery observed in this case. A long duration had passed since the onset of foot drop, during which marked disuse atrophy of the tibialis anterior muscle and a significant reduction in ankle function were observed. NMES has been reported to partially restore neuromuscular function in disused muscles [12]. Electrical stimulation has also been shown to promote neural plasticity and improve motor function [13]. Although electromyographic evaluation could not be performed, the clinical improvements observed suggest that NMES may have contributed to ankle function recovery. The device used in this case delivered HVPC, which is characterized by high voltage and a short pulse width, helping reduce discomfort caused by skin irritation [14]. In conventional electrical stimulation therapy, pain or discomfort may occur even at low stimulation intensities [15], sometimes making adequate treatment intensity difficult to achieve. Since the effectiveness of NMES depends on stimulation intensity [16], inadequate outcomes may result when pain limits the applied intensity. In this case, the use of HVPC allowed for high-intensity stimulation without pain, which likely contributed to the observed improvement in ankle function.

The following considers the timing of FES initiation in this case. The patient was able to walk at the time of admission, and contraction of the tibialis anterior muscle was confirmed through electrical stimulation, indicating that FES could have been applicable. However, due to a strong fear of walking and pronounced limping, FES was initially withheld, and NMES was implemented instead. Although previous studies have reported that FES can improve gait speed and balance [17], other reports suggest that repeated voluntary movement accompanied by stimulation-based feedback leads to greater functional improvements [7]. The inclusion of voluntary effort is considered important in enhancing the therapeutic effects of FES [18]. Based on these findings, active patient participation appears to play a key role in maximizing the benefits of FES. In this case, voluntary contraction of the tibialis anterior muscle was not observed in the early stage of admission, and fear of walking was substantial. Therefore, the therapeutic effect of FES might have been limited if introduced during this severely limping phase. NMES was used initially to strengthen the ankle dorsiflexors and reduce fear of walking, which helped improve gait stability. As a result, a staged intervention strategy that began with NMES and introduced FES after sufficient muscle responsiveness had been achieved may have contributed to the effectiveness of FES in this case.

This case report has several limitations. First, the exact cause of foot drop following LSS surgery is unclear. Therefore, it remains uncertain whether the effects of electrical stimulation therapy observed in this case would be similar to those in foot drop caused by conditions other than LSS, for which its effectiveness has already been demonstrated. Second, the degree to which electrical stimulation therapy contributed to functional improvement is also unclear. Electromyographic evaluation was not performed, making it impossible to objectively confirm the therapeutic effect of electrical stimulation. In addition, the potential influence of general rehabilitation interventions other than NMES and FES cannot be ruled out. To better clarify the effects of electrical stimulation therapy in future cases, it may be necessary to conduct studies using intervention and non-intervention periods for comparison.

## Conclusions

In this case report, electrical stimulation therapy using NMES and FES was applied for residual foot drop following LSS surgery, resulting in improvement in ankle dorsiflexion function, enhancement of walking ability, and reduction in fear of walking. It is considered that the improvement in ankle dorsiflexion function was promoted by NMES using HVPC, and that being able to initiate FES at an appropriate timing facilitated appropriate muscle contraction during walking, which was beneficial. The results of this case suggest the potential of electrical stimulation therapy for foot drop caused by LSS, indicating the need for further case accumulation and investigation.
